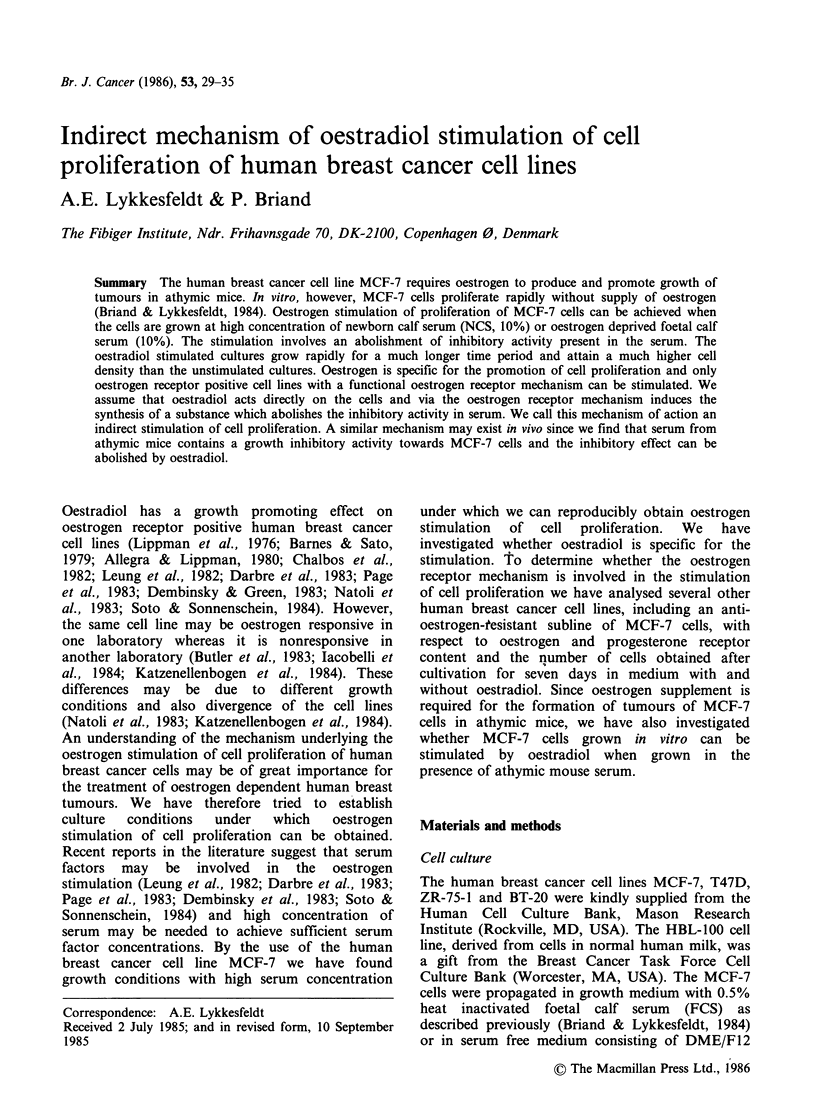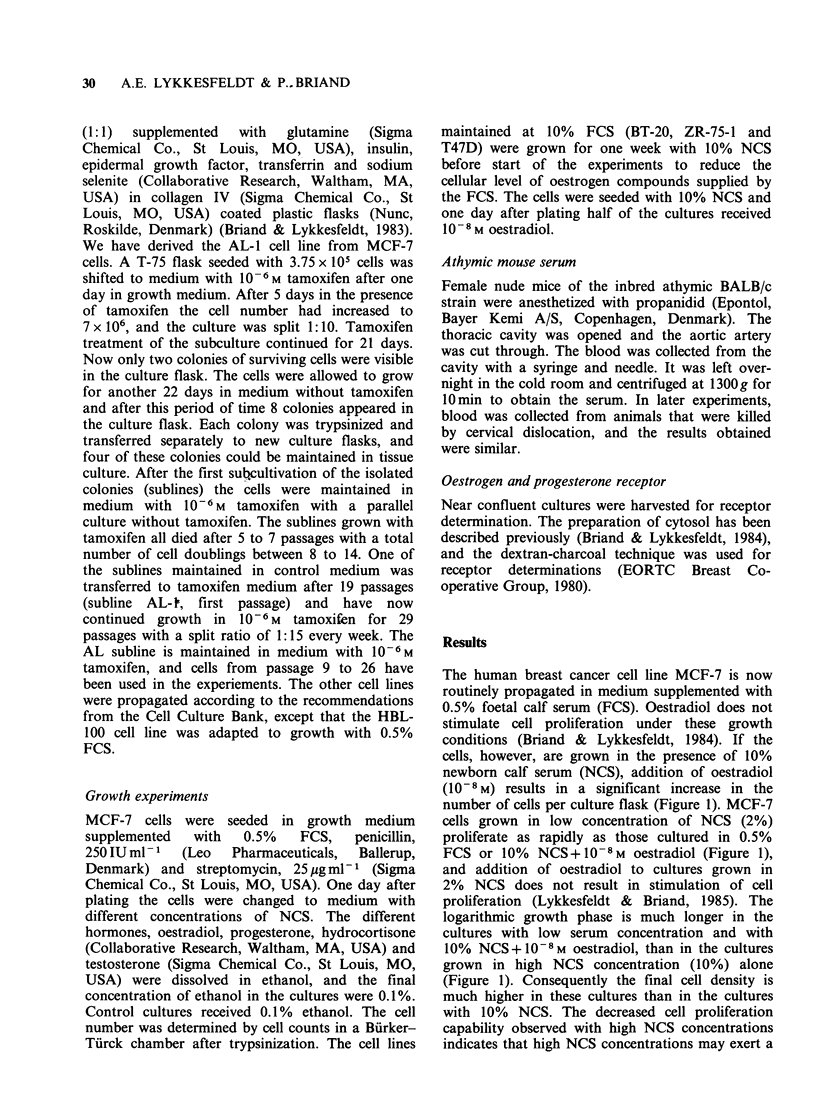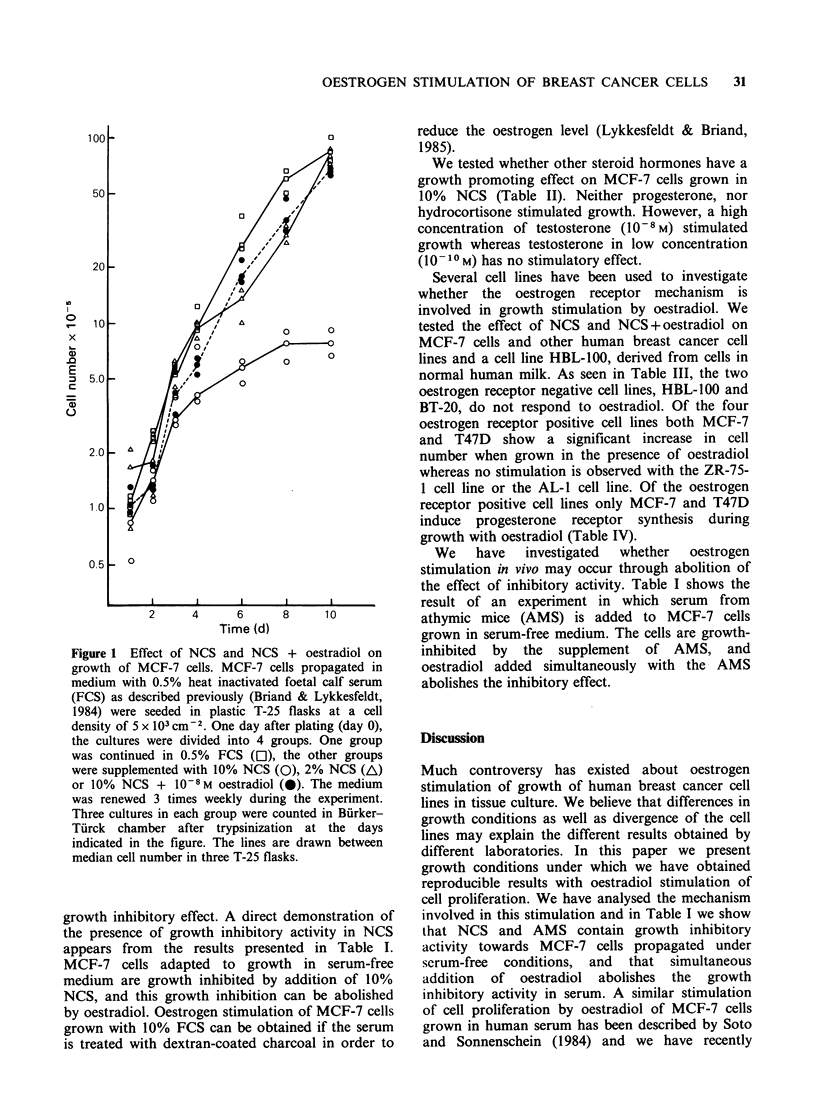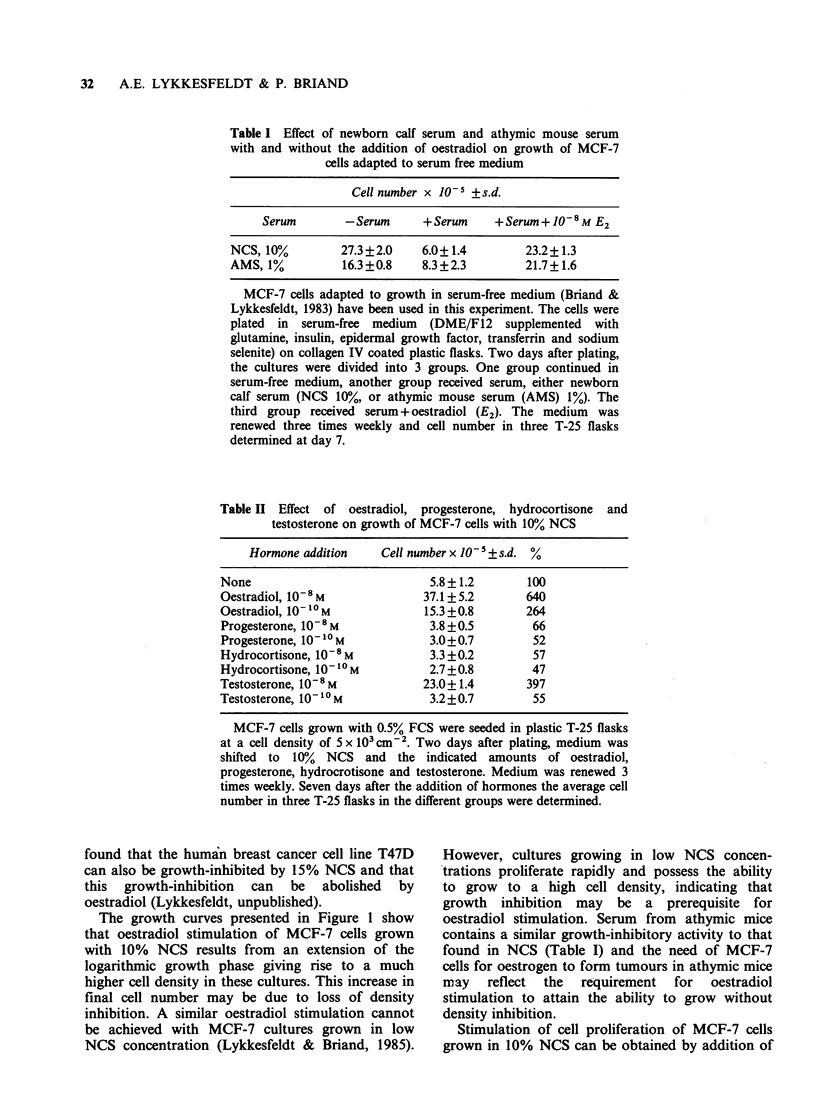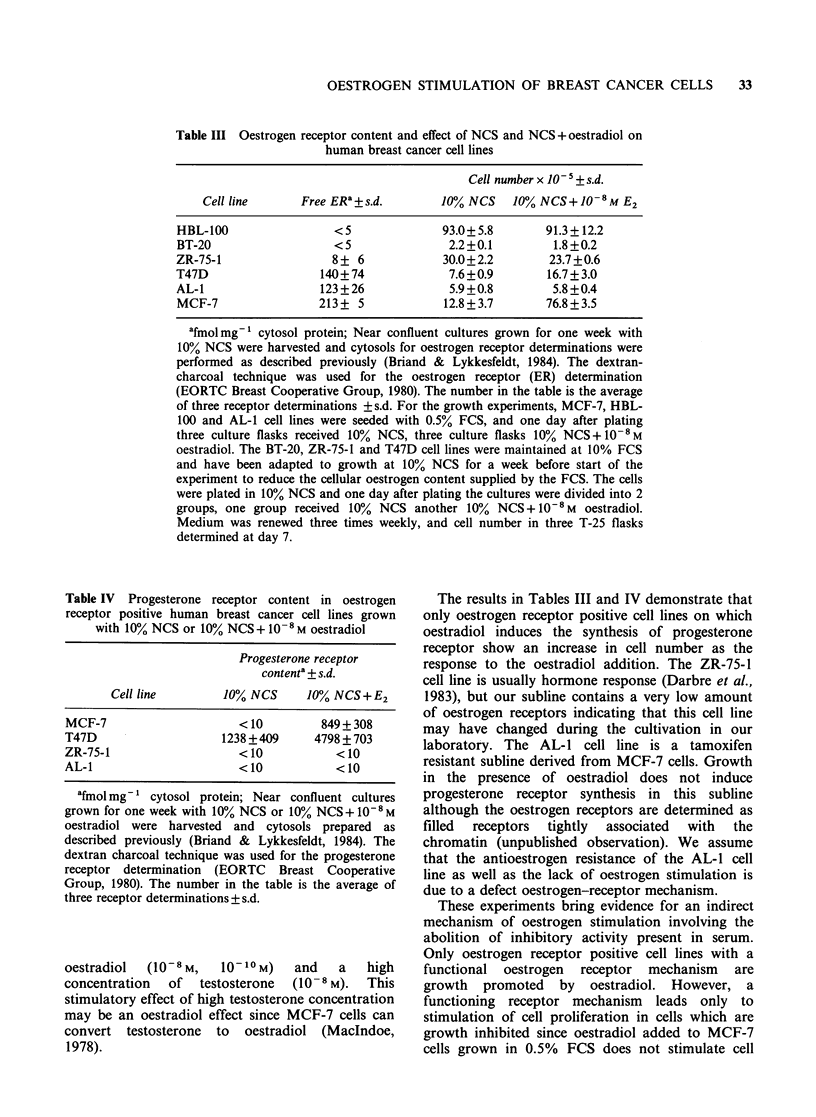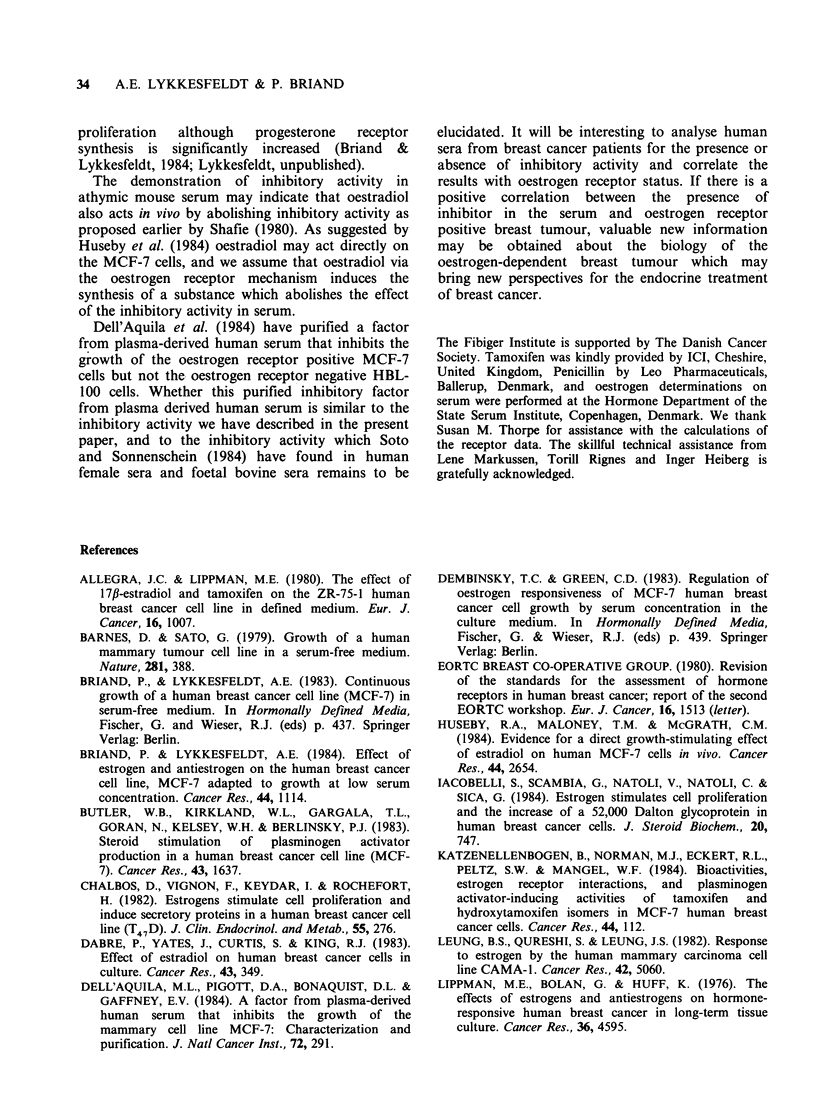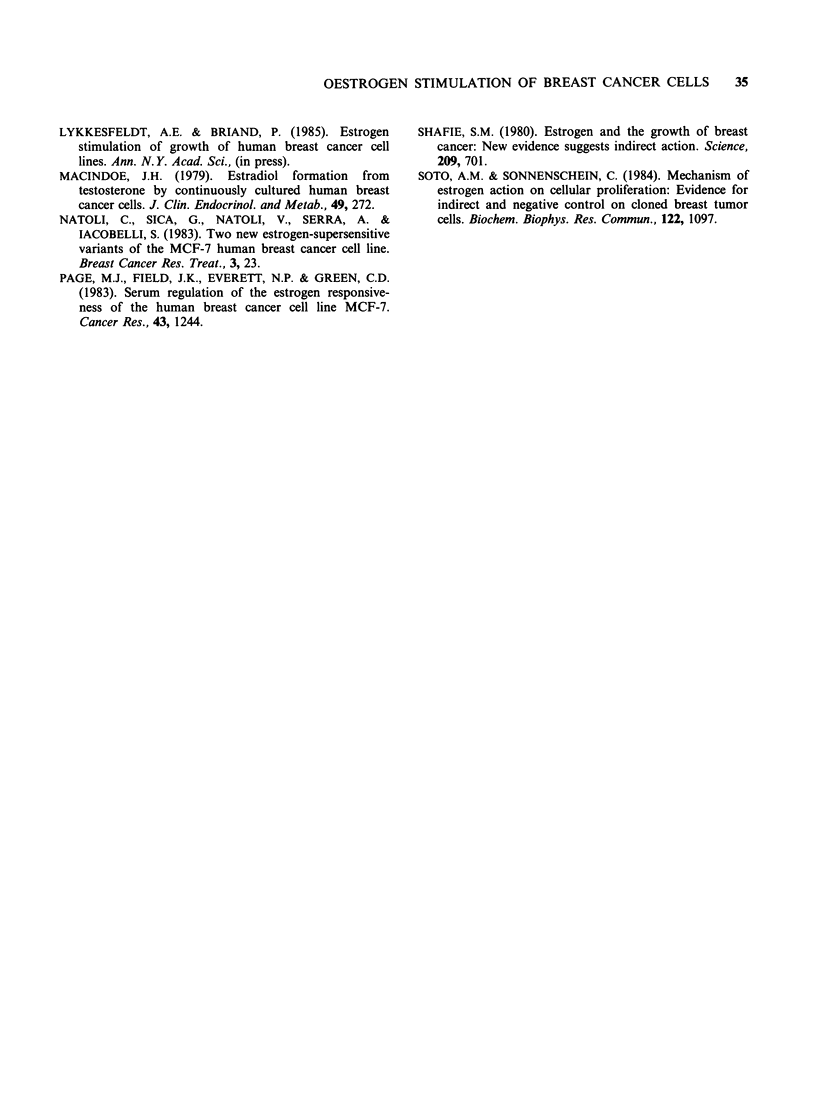# Indirect mechanism of oestradiol stimulation of cell proliferation of human breast cancer cell lines.

**DOI:** 10.1038/bjc.1986.5

**Published:** 1986-01

**Authors:** A. E. Lykkesfeldt, P. Briand

## Abstract

The human breast cancer cell line MCF-7 requires oestrogen to produce and promote growth of tumours in athymic mice. In vitro, however, MCF-7 cells proliferate rapidly without supply of oestrogen (Briand & Lykkesfeldt, 1984). Oestrogen stimulation of proliferation of MCF-7 cells can be achieved when the cells are grown at high concentration of newborn calf serum (NCS, 10%) or oestrogen deprived foetal calf serum (10%). The stimulation involves an abolishment of inhibitory activity present in the serum. The oestradiol stimulated cultures grow rapidly for a much longer time period and attain a much higher cell density than the unstimulated cultures. Oestrogen is specific for the promotion of cell proliferation and only oestrogen receptor positive cell lines with a functional oestrogen receptor mechanism can be stimulated. We assume that oestradiol acts directly on the cells and via the oestrogen receptor mechanism induces the synthesis of a substance which abolishes the inhibitory activity in serum. We call this mechanism of action an indirect stimulation of cell proliferation. A similar mechanism may exist in vivo since we find that serum from athymic mice contains a growth inhibitory activity towards MCF-7 cells and the inhibitory effect can be abolished by oestradiol.


*Br. J. Cancer* (1986), **53**, 29–35

### Indirect mechanism of oestradiol stimulation of cell proliferation of human breast cancer cell lines

A.E. Lykkesfeldt & P. Briand

*The Fibiger Institute, Ndr. Frihavnsgade 70, DK-2100, Copenhagen Ø, Denmark*

**Summary** The human breast cancer cell line MCF-7 requires oestrogen to produce and promote growth of tumours in athymic mice. *In vitro,* however, MCF-7 cells proliferate rapidly without supply of oestrogen (Briand & Lykkesfeldt, 1984). Oestrogen stimulation of proliferation of MCF-7 cells can be achieved when the cells are grown at high concentration of newborn calf serum (NCS, 10%) or oestrogen deprived foetal calf serum (10%). The stimulation involves an abolishment of inhibitory activity present in the serum. The oestradiol stimulated cultures grow rapidly for a much longer time period and attain a much higher cell density than the unstimulated cultures. Oestrogen is specific for the promotion of cell proliferation and only oestrogen receptor positive cell lines with a functional oestrogen receptor mechanism can be stimulated. We assume that oestradiol acts directly on the cells and via the oestrogen receptor mechanism induces the synthesis of a substance which abolishes the inhibitory activity in serum. We call this mechanism of action an indirect stimulation of cell proliferation. A similar mechanism may exist *in vivo* since we find that serum from athymic mice contains a growth inhibitory activity towards MCF-7 cells and the inhibitory effect can be abolished by oestradiol.

Oestradiol has a growth promoting effect on oestrogen receptor positive human breast cancer cell lines (Lippman *et al.,* 1976; Barnes & Sato, 1979; Allegra & Lippman, 1980; Chalbos *et al.,* 1982; Leung *et al.,* 1982; Darbre *et al.,* 1983; Page *et al.,* 1983; Dembinsky & Green, 1983; Natoli *et al.,* 1983; Soto & Sonnenschein, 1984). However, the same cell line may be oestrogen responsive in one laboratory whereas it is nonresponsive in another laboratory (Butler *et al.,* 1983; Iacobelli *et al.,* 1984; Katzenellenbogen *et al.,* 1984). These differences may be due to different growth conditions and also divergence of the cell lines (Natoli *et al.,* 1983; Katzenellenbogen *et al.,* 1984). An understanding of the mechanism underlying the oestrogen stimulation of cell proliferation of human breast cancer cells may be of great importance for the treatment of oestrogen dependent human breast tumours. We have therefore tried to establish culture conditions under which oestrogen stimulation of cell proliferation can be obtained. Recent reports in the literature suggest that serum factors may be involved in the oestrogen stimulation (Leung *et al.,* 1982; Darbre *et al.,* 1983; Page *et al.,* 1983; Dembinsky *et al.,* 1983; Soto & Sonnenschein, 1984) and high concentration of serum may be needed to achieve sufficient serum factor concentrations. By the use of the human breast cancer cell line MCF-7 we have found growth conditions with high serum concentration under which we can reproducibly obtain oestrogen stimulation of cell proliferation. We have investigated whether oestradiol is specific for the stimulation. To determine whether the oestrogen receptor mechanism is involved in the stimulation of cell proliferation we have analysed several other human breast cancer cell lines, including an anti-oestrogen-resistant subline of MCF-7 cells, with respect to oestrogen and progesterone receptor content and the number of cells obtained after cultivation for seven days in medium with and without oestradiol. Since oestrogen supplement is required for the formation of tumours of MCF-7 cells in athymic mice, we have also investigated whether MCF-7 cells grown *in vitro* can be stimulated by oestradiol when grown in the presence of athymic mouse serum.

Correspondence: A.E. Lykkesfeldt
Received 2 July 1985; and in revised form, 10 September 1985

### Materials and methods

### *Cell culture*

The human breast cancer cell lines MCF-7, T47D, ZR-75-1 and BT-20 were kindly supplied from the Human Cell Culture Bank, Mason Research Institute (Rockville, MD, USA). The HBL-100 cell line, derived from cells in normal human milk, was a gift from the Breast Cancer Task Force Cell Culture Bank (Worcester, MA, USA). The MCF-7 cells were propagated in growth medium with 0.5% heat inactivated foetal calf serum (FCS) as described previously (Briand & Lykkesfeldt, 1984) or in serum free medium consisting of DME/F12

© The Macmillan Press Ltd., 1986

(1:1) supplemented with glutamine (Sigma Chemical Co., St Louis, MO, USA), insulin, epidermal growth factor, transferrin and sodium selenite (Collaborative Research, Waltham, MA, USA) in collagen IV (Sigma Chemical Co., St Louis, MO, USA) coated plastic flasks (Nunc, Roskilde, Denmark) (Briand & Lykkesfeldt, 1983). We have derived the AL-1 cell line from MCF-7 cells. A T-75 flask seeded with $3.75 \times 10^5$ cells was shifted to medium with $10^{-6}$ M tamoxifen after one day in growth medium. After 5 days in the presence of tamoxifen the cell number had increased to $7 \times 10^6$, and the culture was split 1:10. Tamoxifen treatment of the subculture continued for 21 days. Now only two colonies of surviving cells were visible in the culture flask. The cells were allowed to grow for another 22 days in medium without tamoxifen and after this period of time 8 colonies appeared in the culture flask. Each colony was trypsinized and transferred separately to new culture flasks, and four of these colonies could be maintained in tissue culture. After the first subcultivation of the isolated colonies (sublines) the cells were maintained in medium with $10^{-6}$ M tamoxifen with a parallel culture without tamoxifen. The sublines grown with tamoxifen all died after 5 to 7 passages with a total number of cell doublings between 8 to 14. One of the sublines maintained in control medium was transferred to tamoxifen medium after 19 passages (subline AL-1, first passage) and have now continued growth in $10^{-6}$ M tamoxifen for 29 passages with a split ratio of 1:15 every week. The AL subline is maintained in medium with $10^{-6}$ M tamoxifen, and cells from passage 9 to 26 have been used in the experiements. The other cell lines were propagated according to the recommendations from the Cell Culture Bank, except that the HBL-100 cell line was adapted to growth with 0.5% FCS.

*Growth experiments*

MCF-7 cells were seeded in growth medium supplemented with 0.5% FCS, penicillin, 250 IU ml$^{-1}$ (Leo Pharmaceuticals, Ballerup, Denmark) and streptomycin, 25 $\mu$g ml$^{-1}$ (Sigma Chemical Co., St Louis, MO, USA). One day after plating the cells were changed to medium with different concentrations of NCS. The different hormones, oestradiol, progesterone, hydrocortisone (Collaborative Research, Waltham, MA, USA) and testosterone (Sigma Chemical Co., St Louis, MO, USA) were dissolved in ethanol, and the final concentration of ethanol in the cultures were 0.1%. Control cultures received 0.1% ethanol. The cell number was determined by cell counts in a Bürker–Türck chamber after trypsinization. The cell lines maintained at 10% FCS (BT-20, ZR-75-1 and T47D) were grown for one week with 10% NCS before start of the experiments to reduce the cellular level of oestrogen compounds supplied by the FCS. The cells were seeded with 10% NCS and one day after plating half of the cultures received $10^{-8}$ M oestradiol.

*Athymic mouse serum*

Female nude mice of the inbred athymic BALB/c strain were anesthetized with propanidid (Epontol, Bayer Kemi A/S, Copenhagen, Denmark). The thoracic cavity was opened and the aortic artery was cut through. The blood was collected from the cavity with a syringe and needle. It was left overnight in the cold room and centrifuged at 1300 $g$ for 10 min to obtain the serum. In later experiments, blood was collected from animals that were killed by cervical dislocation, and the results obtained were similar.

*Oestrogen and progesterone receptor*

Near confluent cultures were harvested for receptor determination. The preparation of cytosol has been described previously (Briand & Lykkesfeldt, 1984), and the dextran-charcoal technique was used for receptor determinations (EORTC Breast Cooperative Group, 1980).

### Results

The human breast cancer cell line MCF-7 is now routinely propagated in medium supplemented with 0.5% foetal calf serum (FCS). Oestradiol does not stimulate cell proliferation under these growth conditions (Briand & Lykkesfeldt, 1984). If the cells, however, are grown in the presence of 10% newborn calf serum (NCS), addition of oestradiol ($10^{-8}$ M) results in a significant increase in the number of cells per culture flask (Figure 1). MCF-7 cells grown in low concentration of NCS (2%) proliferate as rapidly as those cultured in 0.5% FCS or 10% NCS + $10^{-8}$ M oestradiol (Figure 1), and addition of oestradiol to cultures grown in 2% NCS does not result in stimulation of cell proliferation (Lykkesfeldt & Briand, 1985). The logarithmic growth phase is much longer in the cultures with low serum concentration and with 10% NCS + $10^{-8}$ M oestradiol, than in the cultures grown in high NCS concentration (10%) alone (Figure 1). Consequently the final cell density is much higher in these cultures than in the cultures with 10% NCS. The decreased cell proliferation capability observed with high NCS concentrations indicates that high NCS concentrations may exert a

**Figure 1** Effect of NCS and NCS + oestradiol on growth of MCF-7 cells. MCF-7 cells propagated in medium with 0.5% heat inactivated foetal calf serum (FCS) as described previously (Briand & Lykkesfeldt, 1984) were seeded in plastic T-25 flasks at a cell density of $5 \times 10^3 cm^{-2}$. One day after plating (day 0), the cultures were divided into 4 groups. One group was continued in 0.5% FCS (□), the other groups were supplemented with 10% NCS (○), 2% NCS (△) or 10% NCS + $10^{-8}$ M oestradiol (●). The medium was renewed 3 times weekly during the experiment. Three cultures in each group were counted in Bürker–Türck chamber after trypsinization at the days indicated in the figure. The lines are drawn between median cell number in three T-25 flasks.

growth inhibitory effect. A direct demonstration of the presence of growth inhibitory activity in NCS appears from the results presented in Table I. MCF-7 cells adapted to growth in serum-free medium are growth inhibited by addition of 10% NCS, and this growth inhibition can be abolished by oestradiol. Oestrogen stimulation of MCF-7 cells grown with 10% FCS can be obtained if the serum is treated with dextran-coated charcoal in order to reduce the oestrogen level (Lykkesfeldt & Briand, 1985).

We tested whether other steroid hormones have a growth promoting effect on MCF-7 cells grown in 10% NCS (Table II). Neither progesterone, nor hydrocortisone stimulated growth. However, a high concentration of testosterone ($10^{-8}$ M) stimulated growth whereas testosterone in low concentration ($10^{-10}$ M) has no stimulatory effect.

Several cell lines have been used to investigate whether the oestrogen receptor mechanism is involved in growth stimulation by oestradiol. We tested the effect of NCS and NCS + oestradiol on MCF-7 cells and other human breast cancer cell lines and a cell line HBL-100, derived from cells in normal human milk. As seen in Table III, the two oestrogen receptor negative cell lines, HBL-100 and BT-20, do not respond to oestradiol. Of the four oestrogen receptor positive cell lines both MCF-7 and T47D show a significant increase in cell number when grown in the presence of oestradiol whereas no stimulation is observed with the ZR-75-1 cell line or the AL-1 cell line. Of the oestrogen receptor positive cell lines only MCF-7 and T47D induce progesterone receptor synthesis during growth with oestradiol (Table IV).

We have investigated whether oestrogen stimulation *in vivo* may occur through abolition of the effect of inhibitory activity. Table I shows the result of an experiment in which serum from athymic mice (AMS) is added to MCF-7 cells grown in serum-free medium. The cells are growth-inhibited by the supplement of AMS, and oestradiol added simultaneously with the AMS abolishes the inhibitory effect.

### Discussion

Much controversy has existed about oestrogen stimulation of growth of human breast cancer cell lines in tissue culture. We believe that differences in growth conditions as well as divergence of the cell lines may explain the different results obtained by different laboratories. In this paper we present growth conditions under which we have obtained reproducible results with oestradiol stimulation of cell proliferation. We have analysed the mechanism involved in this stimulation and in Table I we show that NCS and AMS contain growth inhibitory activity towards MCF-7 cells propagated under serum-free conditions, and that simultaneous addition of oestradiol abolishes the growth inhibitory activity in serum. A similar stimulation of cell proliferation by oestradiol of MCF-7 cells grown in human serum has been described by Soto and Sonnenschein (1984) and we have recently

**Table I** Effect of newborn calf serum and athymic mouse serum with and without the addition of oestradiol on growth of MCF-7 cells adapted to serum free medium

| | *Cell number* $\times$ *$10^{-5}$* $\pm$*s.d.* | | |
|---|---|---|---|
| *Serum* | *−Serum* | *+Serum* | *+Serum+$10^{-8}$* M *$E_2$* |
| NCS, 10% | 27.3±2.0 | 6.0±1.4 | 23.2±1.3 |
| AMS, 1% | 16.3±0.8 | 8.3±2.3 | 21.7±1.6 |

MCF-7 cells adapted to growth in serum-free medium (Briand & Lykkesfeldt, 1983) have been used in this experiment. The cells were plated in serum-free medium (DME/F12 supplemented with glutamine, insulin, epidermal growth factor, transferrin and sodium selenite) on collagen IV coated plastic flasks. Two days after plating, the cultures were divided into 3 groups. One group continued in serum-free medium, another group received serum, either newborn calf serum (NCS 10%, or athymic mouse serum (AMS) 1%). The third group received serum+oestradiol ($E_2$). The medium was renewed three times weekly and cell number in three T-25 flasks determined at day 7.

**Table II** Effect of oestradiol, progesterone, hydrocortisone and testosterone on growth of MCF-7 cells with 10% NCS

| *Hormone addition* | *Cell number* $\times$ *$10^{-5}$* $\pm$*s.d.* | % |
|---|---|---|
| None | 5.8±1.2 | 100 |
| Oestradiol, $10^{-8}$ M | 37.1±5.2 | 640 |
| Oestradiol, $10^{-10}$ M | 15.3±0.8 | 264 |
| Progesterone, $10^{-8}$ M | 3.8±0.5 | 66 |
| Progesterone, $10^{-10}$ M | 3.0±0.7 | 52 |
| Hydrocortisone, $10^{-8}$ M | 3.3±0.2 | 57 |
| Hydrocortisone, $10^{-10}$ M | 2.7±0.8 | 47 |
| Testosterone, $10^{-8}$ M | 23.0±1.4 | 397 |
| Testosterone, $10^{-10}$ M | 3.2±0.7 | 55 |

MCF-7 cells grown with 0.5% FCS were seeded in plastic T-25 flasks at a cell density of $5 \times 10^3 cm^{-2}$. Two days after plating, medium was shifted to 10% NCS and the indicated amounts of oestradiol, progesterone, hydrocrotisone and testosterone. Medium was renewed 3 times weekly. Seven days after the addition of hormones the average cell number in three T-25 flasks in the different groups were determined.

found that the human breast cancer cell line T47D can also be growth-inhibited by 15% NCS and that this growth-inhibition can be abolished by oestradiol (Lykkesfeldt, unpublished).

The growth curves presented in Figure 1 show that oestradiol stimulation of MCF-7 cells grown with 10% NCS results from an extension of the logarithmic growth phase giving rise to a much higher cell density in these cultures. This increase in final cell number may be due to loss of density inhibition. A similar oestradiol stimulation cannot be achieved with MCF-7 cultures grown in low NCS concentration (Lykkesfeldt & Briand, 1985). However, cultures growing in low NCS concentrations proliferate rapidly and possess the ability to grow to a high cell density, indicating that growth inhibition may be a prerequisite for oestradiol stimulation. Serum from athymic mice contains a similar growth-inhibitory activity to that found in NCS (Table I) and the need of MCF-7 cells for oestrogen to form tumours in athymic mice may reflect the requirement for oestradiol stimulation to attain the ability to grow without density inhibition.

Stimulation of cell proliferation of MCF-7 cells grown in 10% NCS can be obtained by addition of

**Table III** Oestrogen receptor content and effect of NCS and NCS+oestradiol on human breast cancer cell lines

| *Cell line* | *Free ER*[a] $\pm$ *s.d.* | *Cell number* $\times 10^{-5} \pm$ *s.d.* 10% NCS | 10% NCS + $10^{-8}$ M $E_2$ |
|---|---|---|---|
| HBL-100 | <5 | 93.0±5.8 | 91.3±12.2 |
| BT-20 | <5 | 2.2±0.1 | 1.8±0.2 |
| ZR-75-1 | 8± 6 | 30.0±2.2 | 23.7±0.6 |
| T47D | 140±74 | 7.6±0.9 | 16.7±3.0 |
| AL-1 | 123±26 | 5.9±0.8 | 5.8±0.4 |
| MCF-7 | 213± 5 | 12.8±3.7 | 76.8±3.5 |

[a]fmol mg$^{-1}$ cytosol protein; Near confluent cultures grown for one week with 10% NCS were harvested and cytosols for oestrogen receptor determinations were performed as described previously (Briand & Lykkesfeldt, 1984). The dextran-charcoal technique was used for the oestrogen receptor (ER) determination (EORTC Breast Cooperative Group, 1980). The number in the table is the average of three receptor determinations ±s.d. For the growth experiments, MCF-7, HBL-100 and AL-1 cell lines were seeded with 0.5% FCS, and one day after plating three culture flasks received 10% NCS, three culture flasks 10% NCS+$10^{-8}$M oestradiol. The BT-20, ZR-75-1 and T47D cell lines were maintained at 10% FCS and have been adapted to growth at 10% NCS for a week before start of the experiment to reduce the cellular oestrogen content supplied by the FCS. The cells were plated in 10% NCS and one day after plating the cultures were divided into 2 groups, one group received 10% NCS another 10% NCS+$10^{-8}$M oestradiol. Medium was renewed three times weekly, and cell number in three T-25 flasks determined at day 7.

**Table IV** Progesterone receptor content in oestrogen receptor positive human breast cancer cell lines grown with 10% NCS or 10% NCS+$10^{-8}$M oestradiol

| *Cell line* | *Progesterone receptor content*[a] $\pm$ *s.d.* 10% NCS | 10% NCS + $E_2$ |
|---|---|---|
| MCF-7 | <10 | 849±308 |
| T47D | 1238±409 | 4798±703 |
| ZR-75-1 | <10 | <10 |
| AL-1 | <10 | <10 |

[a]fmol mg$^{-1}$ cytosol protein; Near confluent cultures grown for one week with 10% NCS or 10% NCS+$10^{-8}$M oestradiol were harvested and cytosols prepared as described previously (Briand & Lykkesfeldt, 1984). The dextran charcoal technique was used for the progesterone receptor determination (EORTC Breast Cooperative Group, 1980). The number in the table is the average of three receptor determinations±s.d.

oestradiol ($10^{-8}$M, $10^{-10}$M) and a high concentration of testosterone ($10^{-8}$M). This stimulatory effect of high testosterone concentration may be an oestradiol effect since MCF-7 cells can convert testosterone to oestradiol (MacIndoe, 1978).

The results in Tables III and IV demonstrate that only oestrogen receptor positive cell lines on which oestradiol induces the synthesis of progesterone receptor show an increase in cell number as the response to the oestradiol addition. The ZR-75-1 cell line is usually hormone response (Darbre *et al.*, 1983), but our subline contains a very low amount of oestrogen receptors indicating that this cell line may have changed during the cultivation in our laboratory. The AL-1 cell line is a tamoxifen resistant subline derived from MCF-7 cells. Growth in the presence of oestradiol does not induce progesterone receptor synthesis in this subline although the oestrogen receptors are determined as filled receptors tightly associated with the chromatin (unpublished observation). We assume that the antioestrogen resistance of the AL-1 cell line as well as the lack of oestrogen stimulation is due to a defect oestrogen–receptor mechanism.

These experiments bring evidence for an indirect mechanism of oestrogen stimulation involving the abolition of inhibitory activity present in serum. Only oestrogen receptor positive cell lines with a functional oestrogen receptor mechanism are growth promoted by oestradiol. However, a functioning receptor mechanism leads only to stimulation of cell proliferation in cells which are growth inhibited since oestradiol added to MCF-7 cells grown in 0.5% FCS does not stimulate cell proliferation although progesterone receptor synthesis is significantly increased (Briand & Lykkesfeldt, 1984; Lykkesfeldt, unpublished).

The demonstration of inhibitory activity in athymic mouse serum may indicate that oestradiol also acts *in vivo* by abolishing inhibitory activity as proposed earlier by Shafie (1980). As suggested by Huseby *et al.* (1984) oestradiol may act directly on the MCF-7 cells, and we assume that oestradiol via the oestrogen receptor mechanism induces the synthesis of a substance which abolishes the effect of the inhibitory activity in serum.

Dell'Aquila *et al.* (1984) have purified a factor from plasma-derived human serum that inhibits the growth of the oestrogen receptor positive MCF-7 cells but not the oestrogen receptor negative HBL-100 cells. Whether this purified inhibitory factor from plasma derived human serum is similar to the inhibitory activity we have described in the present paper, and to the inhibitory activity which Soto and Sonnenschein (1984) have found in human female sera and foetal bovine sera remains to be elucidated. It will be interesting to analyse human sera from breast cancer patients for the presence or absence of inhibitory activity and correlate the results with oestrogen receptor status. If there is a positive correlation between the presence of inhibitor in the serum and oestrogen receptor positive breast tumour, valuable new information may be obtained about the biology of the oestrogen-dependent breast tumour which may bring new perspectives for the endocrine treatment of breast cancer.

The Fibiger Institute is supported by The Danish Cancer Society. Tamoxifen was kindly provided by ICI, Cheshire, United Kingdom, Penicillin by Leo Pharmaceuticals, Ballerup, Denmark, and oestrogen determinations on serum were performed at the Hormone Department of the State Serum Institute, Copenhagen, Denmark. We thank Susan M. Thorpe for assistance with the calculations of the receptor data. The skillful technical assistance from Lene Markussen, Torill Rignes and Inger Heiberg is gratefully acknowledged.

### References

ALLEGRA, J.C. & LIPPMAN, M.E. (1980). The effect of 17β-estradiol and tamoxifen on the ZR-75-1 human breast cancer cell line in defined medium. *Eur. J. Cancer*, **16**, 1007.

BARNES, D. & SATO, G. (1979). Growth of a human mammary tumour cell line in a serum-free medium. *Nature*, **281**, 388.

BRIAND, P., & LYKKESFELDT, A.E. (1983). Continuous growth of a human breast cancer cell line (MCF-7) in serum-free medium. In *Hormonally Defined Media*, Fischer, G. and Wieser, R.J. (eds) p. 437. Springer Verlag: Berlin.

BRIAND, P. & LYKKESFELDT, A.E. (1984). Effect of estrogen and antiestrogen on the human breast cancer cell line, MCF-7 adapted to growth at low serum concentration. *Cancer Res.*, **44**, 1114.

BUTLER, W.B., KIRKLAND, W.L., GARGALA, T.L., GORAN, N., KELSEY, W.H. & BERLINSKY, P.J. (1983). Steroid stimulation of plasminogen activator production in a human breast cancer cell line (MCF-7). *Cancer Res.*, **43**, 1637.

CHALBOS, D., VIGNON, F., KEYDAR, I. & ROCHEFORT, H. (1982). Estrogens stimulate cell proliferation and induce secretory proteins in a human breast cancer cell line ($T_{47}D$). *J. Clin. Endocrinol. and Metab.*, **55**, 276.

DABRE, P., YATES, J., CURTIS, S. & KING, R.J. (1983). Effect of estradiol on human breast cancer cells in culture. *Cancer Res.*, **43**, 349.

DELL'AQUILA, M.L., PIGOTT, D.A., BONAQUIST, D.L. & GAFFNEY, E.V. (1984). A factor from plasma-derived human serum that inhibits the growth of the mammary cell line MCF-7: Characterization and purification. *J. Natl Cancer Inst.*, **72**, 291.

DEMBINSKY, T.C. & GREEN, C.D. (1983). Regulation of oestrogen responsiveness of MCF-7 human breast cancer cell growth by serum concentration in the culture medium. In *Hormonally Defined Media*, Fischer, G. & Wieser, R.J. (eds) p. 439. Springer Verlag: Berlin.

EORTC BREAST CO-OPERATIVE GROUP. (1980). Revision of the standards for the assessment of hormone receptors in human breast cancer; report of the second EORTC workshop. *Eur. J. Cancer*, **16**, 1513 (*letter*).

HUSEBY, R.A., MALONEY, T.M. & McGRATH, C.M. (1984). Evidence for a direct growth-stimulating effect of estradiol on human MCF-7 cells *in vivo*. *Cancer Res.*, **44**, 2654.

IACOBELLI, S., SCAMBIA, G., NATOLI, V., NATOLI, C. & SICA, G. (1984). Estrogen stimulates cell proliferation and the increase of a 52,000 Dalton glycoprotein in human breast cancer cells. *J. Steroid Biochem.*, **20**, 747.

KATZENELLENBOGEN, B., NORMAN, M.J., ECKERT, R.L., PELTZ, S.W. & MANGEL, W.F. (1984). Bioactivities, estrogen receptor interactions, and plasminogen activator-inducing activities of tamoxifen and hydroxytamoxifen isomers in MCF-7 human breast cancer cells. *Cancer Res.*, **44**, 112.

LEUNG, B.S., QURESHI, S. & LEUNG, J.S. (1982). Response to estrogen by the human mammary carcinoma cell line CAMA-1. *Cancer Res.*, **42**, 5060.

LIPPMAN, M.E., BOLAN, G. & HUFF, K. (1976). The effects of estrogens and antiestrogens on hormone-responsive human breast cancer in long-term tissue culture. *Cancer Res.*, **36**, 4595.

LYKKESFELDT, A.E. & BRIAND, P. (1985). Estrogen stimulation of growth of human breast cancer cell lines. *Ann. N.Y. Acad. Sci.*, (in press).

MACINDOE, J.H. (1979). Estradiol formation from testosterone by continuously cultured human breast cancer cells. *J. Clin. Endocrinol. and Metab.*, **49**, 272.

NATOLI, C., SICA, G., NATOLI, V., SERRA, A. & IACOBELLI, S. (1983). Two new estrogen-supersensitive variants of the MCF-7 human breast cancer cell line. *Breast Cancer Res. Treat.*, **3**, 23.

PAGE, M.J., FIELD, J.K., EVERETT, N.P. & GREEN, C.D. (1983). Serum regulation of the estrogen responsiveness of the human breast cancer cell line MCF-7. *Cancer Res.*, **43**, 1244.

SHAFIE, S.M. (1980). Estrogen and the growth of breast cancer: New evidence suggests indirect action. *Science*, **209**, 701.

SOTO, A.M. & SONNENSCHEIN, C. (1984). Mechanism of estrogen action on cellular proliferation: Evidence for indirect and negative control on cloned breast tumor cells. *Biochem. Biophys. Res. Commun.*, **122**, 1097.